# Transcriptome-wide characterization of candidate genes for improving the water use efficiency of energy crops grown on semiarid land

**DOI:** 10.1093/jxb/erv353

**Published:** 2015-07-13

**Authors:** Yangyang Fan, Qian Wang, Lifang Kang, Wei Liu, Qin Xu, Shilai Xing, Chengcheng Tao, Zhihong Song, Caiyun Zhu, Cong Lin, Juan Yan, Jianqiang Li, Tao Sang

**Affiliations:** ^1^Key Laboratory of Plant Resources and Beijing Botanical Garden, Institute of Botany, Chinese Academy of Sciences, Beijing 100093, China; ^2^State Key Laboratory of Systematic and Evolutionary Botany, Institute of Botany, Chinese Academy of Sciences, Beijing 100093, China; ^3^Key Laboratory of Plant Germplasm Enhancement and Specialty Agriculture, Wuhan Botanical Garden, Chinese Academy of Sciences, Wuhan, Hubei 430074, China; ^4^University of Chinese Academy of Sciences, Beijing 100049, China

**Keywords:** Abiotic stress, adaptation, genetic and environmental interaction, *Miscanthus lutarioriparius*, RNA-Seq, water use efficiency.

## Abstract

Understanding the genetic basis of water use efficiency (WUE) and its roles in plant adaptation to a drought environment is essential for the production of second-generation energy crops in water-deficit marginal land. In this study, RNA-Seq and WUE measurements were performed for 78 individuals of *Miscanthus lutarioriparius* grown in two common gardens, one located in warm and wet Central China near the native habitats of the species and the other located in the semiarid Loess Plateau, the domestication site of the energy crop. The field measurements showed that WUE of *M*. *lutarioriparius* in the semiarid location was significantly higher than that in the wet location. A matrix correlation analysis was conducted between gene expression levels and WUE to identify candidate genes involved in the improvement of WUE from the native to the domestication site. A total of 48 candidate genes were identified and assigned to functional categories, including photosynthesis, stomatal regulation, protein metabolism, and abiotic stress responses. Of these genes, nearly 73% were up-regulated in the semiarid site. It was also found that the relatively high expression variation of the WUE-related genes was affected to a larger extent by environment than by genetic variation. The study demonstrates that transcriptome-wide correlation between physiological phenotypes and expression levels offers an effective means for identifying candidate genes involved in the adaptation to environmental changes.

## Highlight

A matrix correlation analysis between transcriptome-wide expression levels and water use efficiency of *Miscanthus lutarioriparius* identified candidate genes facilitating adaptation of the energy crop to semiarid marginal land.

## Introduction

Drought or water deficiency is one of the most significant abiotic stresses that negatively impacts on plant survival, growth, and productivity (Cattivelli *et al.*, 2008). Decreasing water availability, as a result of increasing industrialization and continuing climate change poses a growing threat to sustainable agriculture (Boyer, 1982; Ings *et al.*, 2013). Water use efficiency (WUE), a drought-adaptive trait that balances carbon assimilated per unit of water transpired, has been linked to drought resistance and higher yields (Wallace, 2000; Tardieu, 2012). WUE is a particularly important factor for dedicated energy crops that are established on marginal land for lignocellulosic feedstock production (Zhang *et al.*, 2011; Suyker and Verma, 2012). Therefore, unravelling the genetic basis of the WUE of energy crops under water deficit conditions holds the potential for the development and improvement of energy crops.

The giant C_4_ grasses from the genus *Miscanthus* have been identified as a valuable germplasm source for second-generation energy crops (Clifton-Brown and Lewandowski, 2000; Carroll and Somerville, 2009; Somerville *et al.*, 2010; Sang, 2011; Feltus and Vandenbrink, 2012; Robson *et al.*, 2013). *Miscanthus lutarioriparius*, an endemic species to central China, produces the highest biomass among the wild *Miscanthus* species and is also capable of high carbon sequestration and effective soil restoration in eroded regions (Chen and Renvoize, 2006; Sang and Zhu, 2011; Mi *et al.*, 2014). When transplanted to the semiarid Loess Plateau where the annual precipitation is less than half of that in its native habitats, it showed higher WUE and produced even higher biomass than in its native habitats (Liu *et al.*, 2012, 2014; Liu and Sang, 2013; Yan *et al.*, 2012, 2015). Whereas it is evident that WUE is of great importance for developing the energy crop in this area, the genetic mechanisms underlying the improvement of WUE in *M*. *lutarioriparius* remains unknown.

A number of studies have previously been devoted to identifying WUE-related loci or genes based on quantitative trait locus (QTL) mapping in various plant species (Martin *et al.*, 1989; Mian *et al.*, 1996; Thumma *et al.*, 2001; Teulat *et al.*, 2002; Hall *et al.*, 2005; Hausmann *et al.*, 2005; Juenger *et al.*, 2005; Masle *et al.*, 2005; de Miguel *et al.*, 2014). For example, *ERECTA*, a leucine-rich repeat receptor-like kinase (LRR-RLK), was the first published gene regulating WUE that has been identified by QTL mapping (Masle *et al.*, 2005).

Microarray analysis was a widely used transcriptomic technique for identifying candidate genes related to drought resistance and WUE in crops and plants with genome sequences, such as *Triticum aestivum* L, *Oryza sativa*, and *Arabidopsis* (Xue *et al.*, 2006; Karaba *et al.*, 2007). The expression profiling analysis performed on wheat revealed that 11 genes are positively correlated with high WUE, measured as carbon isotope discrimination (Xue *et al.*, 2006). The overexpression of the *Arabidopsis HARDY* gene in rice improved WUE by enhancing photosynthetic assimilation and reducing transpiration (Karaba *et al.*, 2007). These studies suggested that these differentially expressed genes were candidates underlying WUE or targets for investigating expression quantitative trait loci (eQTLs). With the development of next-generation sequencing, RNA-Seq has become a powerful tool for the comparative analyses of gene expression which is also applicable to study systems without a reference genome sequence (Martin and Wang, 2011; Zhang *et al.*, 2011; Xu *et al.* 2015).

In this study, RNA-Seq data of 78 individuals of *M*. *lutarioriparius* were compared and a correlation analysis was conducted between gene expression patterns and field-measured WUE. The 78 individuals were collected from the 14 populations of the species and planted in two experimental fields, one near its native habitats in Jiangxia of Hubei Province (JH) and the other in the target domestication site, Qingyang of Gansu Province (QG) located in the Loess Plateau of China (Yan *et al.*, 2012). The leaf-level WUE of these individuals was measured in both fields. To investigate the genetic basis of WUE changes from the native to the domestication site, a method of matrix correlation analysis was developed and candidate genes presumably associated with WUE were identified. These results have provided new insights into the adaptive mechanisms of the energy crops to the semiarid region and may help to speed up energy crop domestication.

## Materials and methods

### Plant materials

Mature seeds of 14 populations of *M*. *lutarioriparius* were collected in October and November 2008 and planted in 2009 in the experimental fields at Jiangxia in Hubei Province (JH) near its native habitats and at Qingyang in Gansu Province (QG) located in the Loess Plateau (Yan *et al.*, 2012). The altitudes of JH and QG were 45 m and 1 258 m, respectively. The average annual temperature was 16.7 °C and the average annual precipitation was 1 319mm in JH, while it was 9.3 °C and 556.5mm in QG, respectively. In this study, 39 individuals of *M*. *lutarioriparius* at each site were sampled from 14 populations taken at random (see Supplementary Table S1 at *JXB* online). The individuals from one site were considered to be one large population because no distinct population structure was detected for either site (Xu *et al.*, 2015).

The samples were kept in liquid nitrogen and brought to the laboratory for RNA isolation. The transcriptome of those leaf samples were sequenced using Illumina HiSeq 2000. The reference transcriptome that included 18 503 unigenes of *M*. *lutarioriparius* was assembled via Population RNA-Seq to Assemble Reference Transcriptome (PopART) as described by Xu *et al.* (2015). The expression level of each sample was estimated as FPKM (fragments per kilobase of exon per million fragments mapped) with Cufflinks v2.0.2 (Trapnell *et al.*, 2010; Xu *et al.*, 2015). The transcriptome coverage per sample, after filtering for read depth, ranged from 41.2% to 74.7%, with an average of 60.4%. Moreover, the sequencing depth was saturated when the number of 80bp reads of an individual used for assembly, reached about 40 million. The number of reads for each individual is presented in Supplementary Table S1 at *JXB* online. Genes in the reference transcriptome had an average length of 1 601bp and N50 of 1 871bp, of which 93.6% ranged from 500–5 000bp [see Table S2 in Xu *et al.* (2015) for details]. The raw sequence data are available at NCBI’s Short Read Archive under three BioProjects, PRJNA227191, PRJNA227195, and PRJNA226258.

### Gas exchange measurements

CO_2_ assimilation rate (*A*) and transpiration rate (*E*) were logged using the LI-6400 portable photosynthesis system (LI-COR 6400 XT system; LI-COR, USA). Instantaneous water use efficiencies (WUE) were calculated as the ratio of the CO_2_ assimilation rate to transpiration rate (*A*/*E*) (Polley, 2002). Counting from the top, measurements were conducted on the middle part of the fourth leaf, which was fully expanded, under ambient temperature and photon flux density, while the CO_2_ concentration was maintained at 400 μmol mol^–1^. An infra-red gas analyser (IRGA) was used to reach equilibrium (monitor ΔCO_2_ and ΔH_2_O) every 20min. These measuring processes were conducted between 10.00h and 12.00h. Once the measurements were completed, the leaves were cut off as soon as possible and frozen immediately in liquid nitrogen for subsequent RNA isolation. Because the growing season was one month later in QG than in JH (Yan *et al.*, 2012), the samples were taken around noon on 12 June 2012 in JH and on 13 July 2012 in QG.

### Matrix correlation between WUE and expression data

**Fig. 1.**
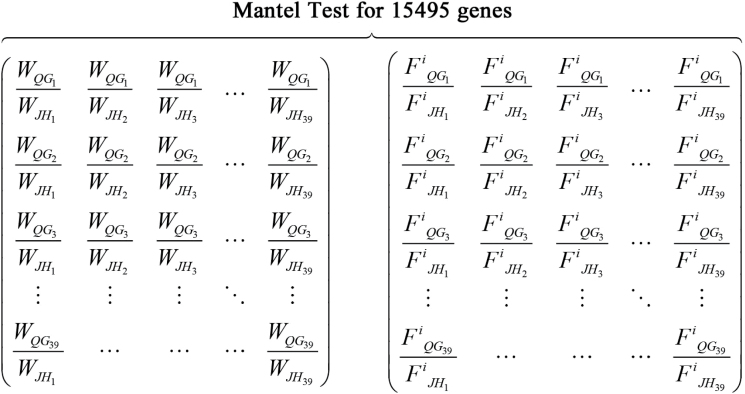
Matrix correlation based on physiological trait and expression data. Water use efficiencies (*W*) ratio and FPKM (*F*) ratio matrices are performed by mantel test via Spearman’s rank correlation method. The mantel test is conducted on 15 495 transcripts of *M*. *lutarioriparius* (*i*=1, 2, …, 15 495). Each correlation is conducted by 10 000 permutations.

As described above, the levels of gene expression for 18 503 transcripts were estimated based on transcript abundance calculated using FPKM. In order to identify the candidate genes responsible for the change of WUE in the new environment, a new method was explored for non-model species without reference genomes. First, genes were weeded out with the median FPKM value of zero and 15 495 genes were left for further analysis. For each gene, the FPKM value of each individual in QG was divided by the value in JH, with all combinations,which resulted in a 39×39 matrix, as it did for the WUE value. The 39×39 WUE matrix was then correlated with each 39×39 FPKM matrix by mantel test using Spearman’s rank correlation method (Fig. 1). The analyses were conducted using vegan package (version 2.0–10) in the R statistical environment release 3.0.2. Thus, a Spearman’s rank correlation coefficient was generated for each gene while using a 10 000 permutation test to assess its statistical significance.

### Functional categorization and expression pattern analysis of candidate genes

The whole transcriptome of *M*. *lutarioriparius* was annotated and a search was made for all the photosynthesis genes of *Zea mays* and *Sorghum* in the KEGG and NCBI databases. Then the DNA sequences of these genes were compared with *M*. *lutarioriparius* transcripts and photosynthesis genes in *M*. *lutarioriparius* were identified. In addition, a detailed functional categorization for candidates was performed using the Nucleotide Basic Local Alignment Search Tool (BlastN) on the National Center for Biotechnology Information (NCBI) non-redundant nucleotide database (Nt). The expectation value (*E* value) was used to determine the most likely result of a query sequence. Only those results with an *E* value lower than 10^–10^ were considered.

Hierarchical cluster analysis was conducted from the expression levels of all candidate genes (mantel test, *P* <0.01), using Pearson’s correlation distance in Multiexperiment Viewer (MEV) 4.9 software (Saeed *et al.*, 2003). All of the absolute FPKM values of each individual in JH and QG were normalized (divided by the standard deviation of the observations). The differentially expressed genes of candidate genes between the two sites were identified using the *t* test (normal distribution) or the Wilcoxon test (non-normal distribution) on expression levels. In order to control the family-wise type I error rate (FWE), the raw *P* values were adjusted using the Benjamini and Hochberg method (1995), which corrected for false discovery rate (FDR).

### Validation of gene expression from RNA-Seq

Eight randomly selected genes were used to validate the expression profiling accuracy of RNA-Seq by quantitative real-time PCR (qPCR). The RNA samples were the same as the ones for RNA-Seq. The qPCR primers for the amplification of targeted genes were designed using Primer Premier 5.0 (Premier Biosoft International). Complementary DNA (cDNA) synthesis was carried out using PrimeScript™ Reverse Transcriptase (Takara). Amplification of cDNA was monitored using a SYBR Premix Ex Taq (Takara) on a StepOne Plus Real-Time PCR system (Life Technologies). Each PCR reaction contained 2 μl of the diluted cDNA, 10 μl Takara SYBR Premix Ex Taq, 6.8 μl of nuclease-free water, and 0.8 μl of the forward and reverse primers (10 mΜ stock) in a 20 μl reaction mixture. The PCR cycling conditions were as follows: 95 °C for 30 s, followed by 40 cycles of 95 °C for 5 s and 60 °C for 40 s. The melting curve was routinely performed after 40 cycles to verify primer specificity. Three technical replicates were analysed for each template to calculate the average and standard deviation of the expression levels. Relative expression levels of target genes among the sampled individuals were determined using the 2^–ΔΔCt^ method with the *β-actin* gene used as the normalizer (Schmittgen *et al.*, 2000).

### Genetic variation of candidate genes

Single nucleotide polymorphisms (SNPs) were identified using SAMtools with default settings as described in Xu *et al.* (2015). Genotypes of all individuals in SNPs within one gene were pooled together as input data for haplotype inferences using PHASE v2.1.1 software which was based on a coalescent model and a hidden Markov model (HMM) method (Stephens and Donnelly, 2003). Nucleotide diversity (π) was calculated for each transcript in JH and QG using custom Perl script according to the method introduced by Nei and Li (1979). In our study, haplotypes were considered as alleles, which were the connection of SNPs within a gene. The genotype of each individual was the best reconstruction of haplotypes with the highest probability. The average gene expression levels were computed for individuals with the same genotype (The minimum number of individuals for each genotype which was compared was not less than three, *n* ≥3.) To quantify the effects of genotype, environment, and genotype-by-environment interaction on the candidate genes with genetic variation, a two-way (genotype and environment) factorial analysis of variance (ANOVA) was performed directly on the transcriptional levels (FPKM) (Anholt and Mackay, 2004; Landry *et al.*, 2006). For each transcript, firstly, an ANOVA was performed in R project (http://www.R-project.org) taking genotype, environment, and their interaction into consideration. In the second step, genes without significant genotype×environment effect were analysed using a model without any interaction effect to identify those genes showing main effects of genotype and growth environment alone.

## Results

### Comparison of water use efficiencies of *M*. *lutarioriparius* between two sites

**Fig. 2.**
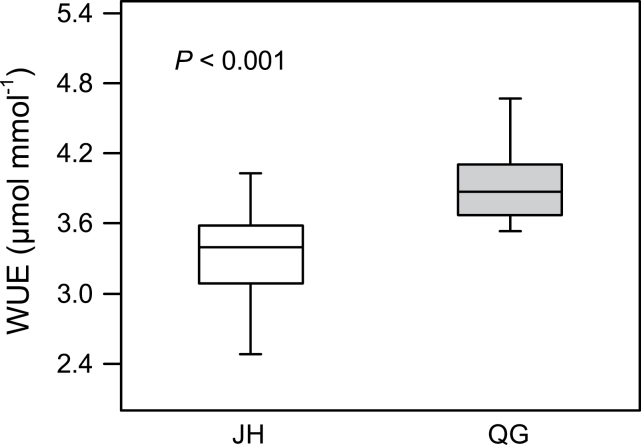
Comparison of water use efficiencies (WUE) of *M*. *lutarioriparius* between two sites. The empty boxplot shows the mean value of WUE in Jiangxia of Hubei Province (JH) and the solid boxplot the mean value of WUE in Qingyang of Gansu Province (QG). The *t* test between the WUE values in JH and QG is examined and the result shows that the WUE value of *M*. *lutarioriparius* is significantly higher in QG than in JH (*P* <0.001).

WUE ranged from 2.484 to 4.027 μmol mmol^–1^ in JH, while it ranged from 2.886 to 4.930 μmol mmol^–1^ in QG. The minimum value of WUE was lower in JH than in QG, and the maximum value of WUE showed the same pattern. In addition, the average WUE in JH and QG were 3.340 and 3.956 μmol mmol^–1^, respectively. In order to detect the differences of WUE between *M*. *lutarioriparius* individuals in JH and QG, an unpaired two-group Student’s *t* test analysis was carried out which showed that the WUE of *M*. *lutarioriparius* individuals was significantly higher in QG than those in JH (*P* <0.001; Fig. 2). The CO_2_ assimilation rate (*A*) and transpiration rate (*E*) were recorded for all individuals in both fields (see Supplementary Fig. S1 at *JXB* online).

### Identification and classification of candidate genes in the *M*. *lutarioriparius* transcriptome

According to the matrix correlation between expression levels and WUE, a total of 48 genes were identified at the *P* <0.01 level and predicted to be candidates for WUE alteration in the new environment. Correlation coefficients (*r*) of these genes ranged from 0.232 to 0.429, revealing their contributions to WUE alteration (Table 1). The detailed functional categorization of candidates was performed on NCBI using BlastN with *E* values lower than 10^–10^ (Table 2).

**Table 1.** Candidate genes of water use efficiency (WUE) identified at the 0.01 level by mantel test

No.	Transcripts	Coefficients of mantel test	*P* value
1	MluLR14810	0.429	0.001
2	MluLR13061	0.363	0.001
3	MluLR17433	0.361	0.001
4	MluLR5439	0.351	0.001
5	MluLR10901	0.348	0.001
6	MluLR18372	0.343	0.001
7	MluLR5367	0.339	0.002
8	MluLR17105	0.318	0.002
9	MluLR16034	0.316	0.004
10	MluLR14116	0.316	0.002
11	MluLR8003	0.313	0.003
12	MluLR15213	0.312	0.003
13	MluLR12315	0.311	0.010
14	MluLR8832	0.309	0.002
15	MluLR17106	0.308	0.004
16	MluLR14298	0.306	0.007
17	MluLR10824	0.305	0.003
18	MluLR16796	0.305	0.005
19	MluLR2994	0.302	0.001
20	MluLR5858	0.300	0.004
21	MluLR14458	0.299	0.001
22	MluLR18370	0.296	0.005
23	MluLR11713	0.293	0.003
24	MluLR3563	0.289	0.002
25	MluLR3628	0.288	0.007
26	MluLR1213	0.288	0.006
27	MluLR17104	0.287	0.001
28	MluLR17108	0.276	0.007
29	MluLR11870	0.276	0.008
30	MluLR9412	0.272	0.002
31	MluLR4945	0.263	0.009
32	MluLR5294	0.261	0.005
33	MluLR8498	0.259	0.007
34	MluLR2876	0.259	0.003
35	MluLR18313	0.257	0.009
36	MluLR7126	0.256	0.010
37	MluLR15163	0.253	0.008
38	MluLR4277	0.252	0.010
39	MluLR420	0.252	0.010
40	MluLR12213	0.251	0.009
41	MluLR12611	0.250	0.008
42	MluLR17402	0.246	0.008
43	MluLR18082	0.242	0.007
44	MluLR15146	0.242	0.006
45	MluLR16886	0.241	0.007
46	MluLR4566	0.235	0.006
47	MluLR17624	0.234	0.010
48	MluLR4148	0.232	0.008

**Table 2.** Functional categorization and putative annotation of candidate genes The annotation and potential functional groups of candidate genes are performed using Nucleotide Basic Local Alignment Search Tool (BlastN) on National Center for Biotechnology Information (NCBI). The abbreviations of annotation and the best species of sequence alignments are enclosed in parentheses and brackets, respectively.

Functional categorization	Transcripts	BlastN	*P* value
Photosynthesis	MluLR14810	Photosystem II reaction centre protein K (PsbK) [*Zea mays*]	0
	MluLR17433	Photosystem II reaction centre protein I (PsbI) [*Zea mays*]	0
	MluLR17106	Photosystem I assembly protein Ycf4 (Ycf4),	
		photosystem I subunit VIII (PsaI) [*Zea mays*]	0
	MluLR16796	Chloroplast envelope membrane protein-like (CemA-like) [*Zea mays*]	1.00E-139
	MluLR17108	Photosystem II reaction centre protein H (PsbH) [*Zea mays*]	0
	MluLR15163	Thioredoxin-like [*Zea mays*]	0
	MluLR4277	Pyruvate orthophosphate dikinase regulatory protein (PDRP) [*Sorghum bicolor*]	0
	MluLR17402	Plastocyanin (petE) [*Zea mays*]	2.00E-100
Stomatal regulation	MluLR8003	Cysteine-rich receptor-like protein kinase 10-like (CRK10-like) [*Oryza brachyantha*]	3.00E-85
	MluLR16886	WRKY transcription factor 4 (WRKY4) [*Zea mays*]	0
	MluLR8832	Hydroxyacid oxidase 1 (HAO1) [*Zea mays*]	0
	MluLR5858	WUSCHEL-related homeobox 14 (WOX14) [*Zea mays*]	0
	MluLR14458	Auxin response factor 4 (ARF4) [*Zea mays*]	0
	MluLR5294	Anion transporter 4 (OAT4) [*Zea mays*]	0
	MluLR2876	Ubiquitin-protein ligase E3 (UBE3) [*Zea mays*]	0
	MluLR7126	Starch synthase II-2 [*Sorghum bicolor*]	0
	MluLR12213	Hexose carrier protein 6 (HEX6) [*Zea mays*]	0
Abiotic stress responses	MluLR13061	Lysine-specific histone demethylase 1 (LSD1) [*Zea mays*]	0
	MluLR16034	Cyclophilin type peptidyl-prolyl cis-trans isomerase (Cyclophilin type PPIase) [*Zea mays*]	0
	MluLR15146	FKBP-type peptidyl-prolyl cis-trans isomerase 4 (FKBP-type PPIase) [*Zea mays*]	0
	MluLR15213	Prolyl 4-hydroxylase alpha-2 subunit (P4HA1) [*Zea mays*]	0
	MluLR18313	18.8kDa class V heat shock protein (HSP18.8) [*Zea mays*]	0
	MluLR4566	DEAD-box ATP-dependent RNA helicase 57-like (RH57-like) [*Zea mays*]	0
	MluLR2994	Alcohol dehydrogenase-like 5-like (ADH5-like) [*Setaria italica*]	0
	MluLR11870	SRG1-like protein [*Setaria italica*]	0
	MluLR4945	Lecithin-cholesterol acyltransferase-like 1-like (LCAT1-like) [*Setaria italica*]	0
	MluLR12315	Methyltransferase-like protein 2-like (Mettl2-like) [*Setaria italica*]	0
	MluLR12611	Methyl-CpG binding domain106 [*Zea mays*]	0
	MluLR17624	RLC retrotransposon [*Saccharum*]	0
Protein metabolism	MluLR10901	Ammonium transporter 2 (AMT2) [*Zea mays*]	0
	MluLR17105	Ribosomal protein S4 [*Zea mays*]	0
	MluLR3628	ORMDL family protein [*Zea mays*]	0
	MluLR17104	Ribosomal protein S16 [*Zea mays*]	4.00E-163
Others	MluLR5439	Hypothetical protein [*Sorghum bicolor*]	0
	MluLR18372	Hypothetical protein [*Sorghum bicolor*]	0
	MluLR5367	Hypothetical protein [*Sorghum bicolor*]	0
	MluLR14116	Hypothetical protein [*Sorghum bicolor*]	0
	MluLR14298	Transcription factor E2F3 [*Zea mays*]	0
	MluLR10824	Hypothetical protein [*Sorghum bicolor*]	0
	MluLR18370	Isoaspartyl peptidase/L-asparaginase 1-like (ASRGL1-like) [*Setaria italica*]	0
	MluLR11713	Unknown [*Saccharum*]	6.00E-160
	MluLR3563	Hypothetical protein [*Sorghum bicolor*]	0
	MluLR1213	Hypothetical protein [*Sorghum bicolor*]	0
	MluLR9412	Unknown [*Zea mays*]	0
	MluLR8498	Unknown [*Zea mays*]	0
	MluLR420	DNA topoisomerase 2-binding protein 1 (TOPBP1) [*Zea mays*]	0
	MluLR18082	Hypothetical protein [*Sorghum bicolor*]	2.00E-157
	MluLR4148	Hypothetical protein [*Sorghum bicolor*]	0

According to the blast, there were eight photosynthesis-related genes among the identified candidates. Five of them encoded proteins involved in the assembly of photosystems I and II and one was involved in the C_4_ pathway. There were nine genes found to be related to stomatal regulation, of which five could regulate abscisic acid (ABA) signal transduction. One gene encoded a homeobox transcription factor regulating stomata density and other three encoded proteins regulating the stomatal movements. Twelve abiotic stress-related genes were also found in the 48 candidate genes. They participated in multiple pathways responding to abiotic stress, including drought stress and salt stress. Four genes were involved in protein metabolism. The remaining genes included 12 with unknown function (Table 2). Of 36 candidate gene with annotated functions, one third are abiotic stress-related, followed by the stomatal regulation-related and the photosynthesis-related genes, each accounting for about one quarter.

### Expression pattern of candidate genes in two sites

**Fig. 3.**
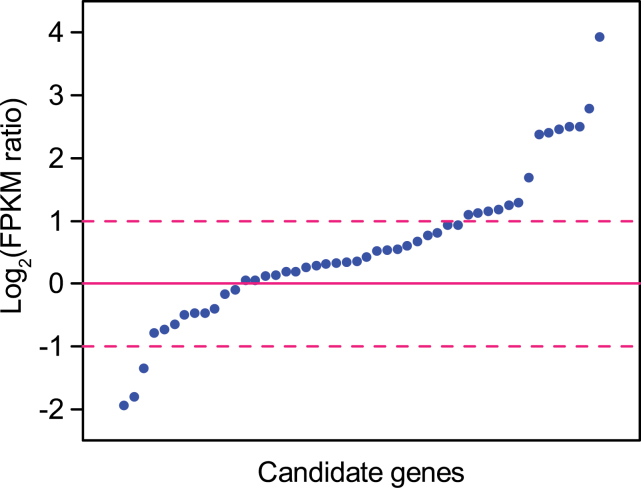
The distribution of gene expression change patterns of the candidate genes. Fold changes of gene expression levels are expressed in log_2_ (FPKM ratio), where the FPKM ratio was calculated as the ratio of FPKM (QG) to FPKM (JH). FPKM (JH) and FPKM (QG) values represent the average expression levels of each transcript in the experimental fields in Jiangxia of Hubei Province (JH) and Qingyang of Gansu Province (QG), respectively. The log-ratios beyond zero represent up-regulated genes, while the ratio of 1 and –1 mean 2-fold up-regulation and down-regulation, respectively.

The fold changes of average expression levels of the candidate genes between the two sites ranged from 0.26 to 15.60 (see Supplementary Table S2 at *JXB* online). Compared with those in JH, 72.92% and 27.08% of the genes were up-regulated and down-regulated in QG, and 29.2% and 6.25% of the candidates were up-regulated and down-regulated more than 2-fold, respectively (Fig. 3). Fourteen genes were up-regulated more than 2-fold including three photosynthesis-related genes, three stomatal regulation-related, and four abiotic stress-related. Three genes down-regulated more than 2-fold belonged to the ‘Other’ functional category.

Fifteen candidates showed significant differentiation on gene expression levels (*P* <0.05; Table 3). Ten were significantly up-regulated, including five photosynthesis-related genes, two stomatal regulation-related, one abiotic stress-related, one protein metabolism-related gene, and one gene in the ‘Others’ functional category. Genes encoding the ORMDL family protein, ASRGL1-like, and three hypothetical proteins were significantly down-regulated. The results showed that 62.5% of photosynthesis-related genes were significantly up-regulated in QG. Considering the significant enhancement of WUE in QG (Fig. 2), the up-regulation of photosynthesis-related genes should have played an important role in regulating WUE upon water deficiency.

**Table 3.** Differentially expressed genes of candidates at the 0.05 level The statistics of the *t* test (normal distribution) or the Wilcoxon test (non-normal distribution) on expression levels was carried out between individuals in JH and QG. *P* values were adjusted using the Benjamini and Hochberg method (1995), which monitored the false discovery rate (FDR). Up-regulated genes represent that the expression levels are higher in QG than in JH, and down-regulated genes show that the expression levels are lower in QG than in JH.

Transcripts	Functional category	Annotation	*P* value
Up-regulated			
MluLR14810	Photosynthesis	Photosystem II reaction centre protein K	4.46E-04
MluLR17433	Photosynthesis	Photosystem II reaction centre I protein I	1.43E-04
MluLR17106	Photosynthesis	Photosystem I assembly protein Ycf4, photosystem I subunit VIII	5.29E-13
MluLR17108	Photosynthesis	Photosystem II reaction centre protein H	2.33E-11
MluLR17402	Photosynthesis	Plastocyanin	3.86E-04
MluLR5294	Stomatal regulation	Anion transporter 4	1.25E-16
MluLR2876	Stomatal regulation	Ubiquitin-protein ligase E3	1.12E-06
MluLR15146	Abiotic stress responses	FKBP-type peptidyl-prolyl cis-trans isomerase 4	8.60E-03
MluLR17105	Protein metabolism	Ribosomal protein S4	2.11E-06
MluLR4945	Others	Hypothetical protein	6.12E-08
Down-regulated			
MluLR3628	Protein metabolism	ORMDL family protein	6.68E-06
MluLR18372	Others	Hypothetical protein	8.79E-11
MluLR18370	Others	Isoaspartyl peptidase/L-asparaginase 1-like	4.46E-15
MluLR3563	Others	Hypothetical protein	5.37E-05
MluLR18102	Others	Hypothetical protein	4.65E-05

**Fig. 4.**
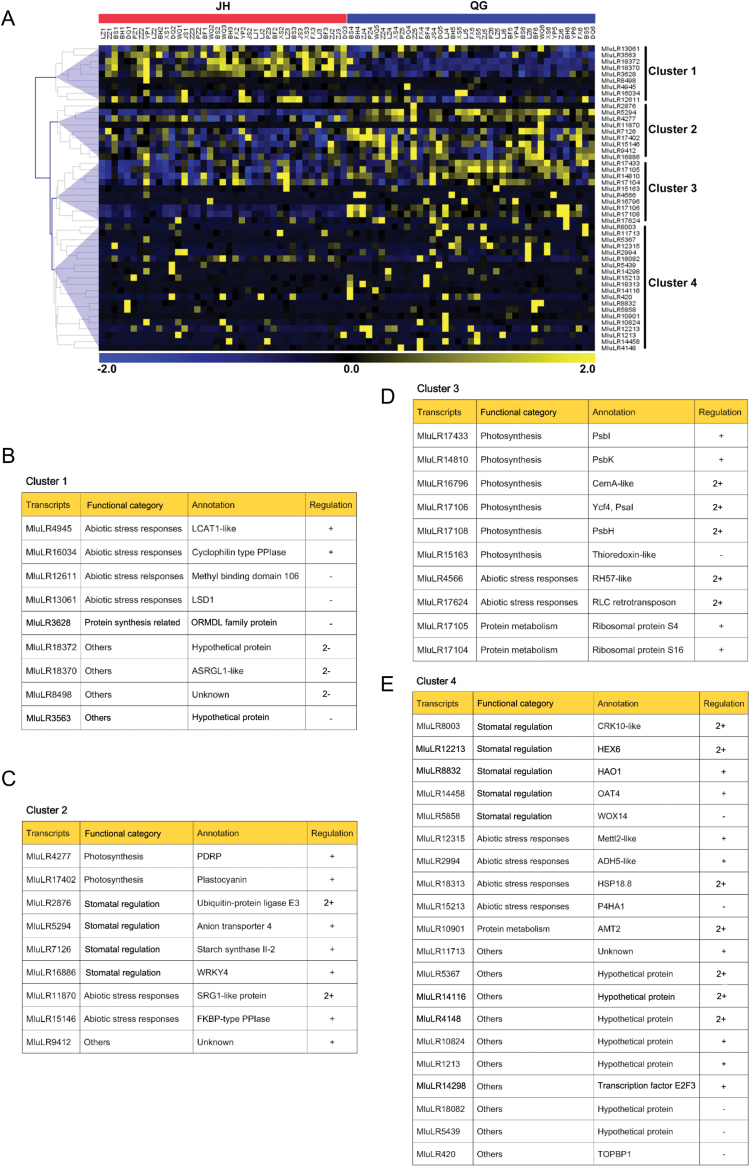
(A) Hierarchical clustering of 48 candidate genes differentially expressed between individuals in JH (left) and QG (right). The normalized gene expression values (FPKM) of candidate genes of each individual are used for the cluster display. The colour scale (representing the normalized gene expression) is shown at the bottom. The genes which share similar expression patterns are divided into four groups, (B) Cluster 1, (C) Cluster 2, (D) Cluster 3, and (E) Cluster 4. For the full names of abbreviations in annotation see Table 2. Up-regulated (+), down-regulated (–), up-regulated more than 2-fold (2+), down-regulated more than 2-fold (2–).

Cluster analysis of the 48 candidate genes with normalized expression levels revealed four major groups (Fig. 4A). Cluster 1 comprised nine genes that were expressed largely at higher levels in JH than in QG (Fig. 4B). It contained four genes related to abiotic stress responses and one gene ‘MluLR3628’ encoding the ORMDL family protein involved in protein folding in the endoplasmic reticulum. The functions of MluLR4945, MluLR16034, MluLR12611, and MluLR13061 were related to abiotic stress and encoded lecithin-cholesterol acyltransferase-like 1 (LCAT1-like), cyclophilin type peptidyl-prolyl *cis*-*trans* isomerase (cyclophilin type PPIase), methyl-CpG binding domain 106 protein, and lysine-specific histone demethylase 1 (LSD1), respectively.

Cluster 2 included nine genes showing higher expression levels in QG than in JH (Fig. 4C). Four genes belonged to the ‘Stomatal regulation-related’ functional category, including genes encoding ubiquitin-protein ligase E3, anion transporter 4, starch synthase II-2, and WRKY transcription factor 4 (WRKY4). Among them, MluLR2876 and MluLR11870 encoding ubiquitin-protein ligase E3 and SRG1-like proteins, respectively, were up-regulated more than 2-fold in QG. The ‘Photosynthesis’ functional category was also represented, including genes encoding PDRP and plastocyanin (petE). In addition, genes encoding SRG1-like protein and FKBP-type peptidyl-prolyl *cis*-*trans* isomerase 4 (FKBP-type PPIase) related to abiotic stress were also clustered in this group.

Cluster 3 contained ten genes that were mostly expressed at higher levels in QG (Fig. 4D). Most of these genes represented the ‘Photosynthesis’ functional category, including those encoding PsbI, PsbK, PsbH, Ycf4/PsaI, CemA-like, and thioredoxin-like proteins. The enrichment in photosynthesis-related genes, with a proportion of 60% in this cluster, suggested a powerful coexpression pattern of photosynthesis-related genes in regulating WUE under the water deficit conditions. Genes encoding CemA-like, Ycf4/PsaI, and PsbH, were up-regulated more than 2-fold in QG. Furthermore, this group included two genes in the chloroplast genome encoding ribosomal protein S4 and S16 that both played crucial roles in protein metabolism. Two genes encoding RH57-like and RLC retrotransposon involved in abiotic stress responses were also clustered in this group and were up-regulated more than 2-fold in QG.

Finally, Cluster 4 comprised 20 genes that were up-regulated in QG compared with lower expression levels in JH (Fig. 4E). Five genes were related to stomatal regulation, including three involved in the response to ABA, which plays a pivotal role in adaptation to drought stress and the production of reactive oxygen species (ROS). The expression levels of genes encoding CRK10 and HEX6 were increased more than two times in QG. MluLR10901 encoding ammonium transporter 2 was up-regulated more than 2-fold in QG, which was subjected to nitrogen regulation (Sohlenkamp *et al.*, 2000). Four genes related to abiotic stress were also identified, including genes encoding methyltransferase-like protein 2-like (Mettl2-like), alcohol dehydrogenase-like 5-like (ADH5-like), prolyl 4-hydroxylase alpha-2 subunit (P4HA1), and 18.8kDa class V heat shock protein (HSP18.8). Among them, HSP18.8 expression was increased more than 2-fold in QG. In addition, ten genes encoding products with unknown function were clustered in this group.

**Fig. 5.**
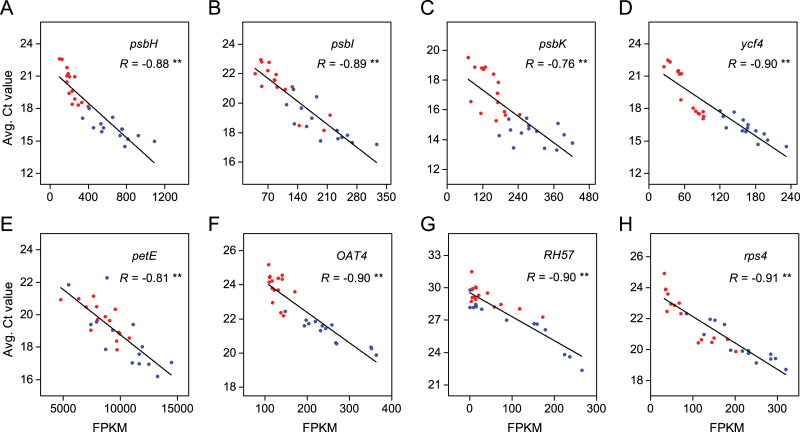
Expression level correlation between RNA-Seq and qPCR. Negative correlation between FPKM values of RNA-Seq and average Ct values of qPCR indicate a consistent estimation of the relative expression levels between the two methods. The graphs (A)–(H) represent the genes: MluLR17108 (*psbH*), MluLR17433 (*psbI*), MluLR14810 (*psbK*), MluLR17106 (*ycf4*), MluLR17402 (*petE*), MluLR5294 (*OAT4*), MluLR4566 (*RH57*), MluLR17105 (*rps4*), respectively. The *R* in the graphs indicates the correlation coefficient. ** represents the significant level (*P* <0.01, Spearman’s rank correlation test). Sequences of PCR primers are given in Table 4. Red and blue dots represent individuals sampled from JH and QG, respectively.

Quantitative real-time PCR was performed by using 15 randomly sampled individuals from each field site for eight genes, *psbH*, *psbI*, *psbK*, *ycf4*, *petE*, *OAT4*, *RH57*, and *rps4* (Table 4). The relative expression levels determined by the two methods were significantly correlated for all eight genes (Spearman’s rank correlation test, *P* <0.01; Fig. 5).

**Table 4.** Primers for quantitative real-time PCR.

Transcripts	Gene name	Primers
MluLR17108	*psbH*	Forward: GACCTAAGCCAAAACGGAC
		Reverse: CGAATAAAGCCATTGCGAC
MluLR17433	*psbI*	Forward: CTTATCTAATGACCCAGGACG
		Reverse: AGAGATGGCTGAGTGGACT
MluLR14810	*psbK*	Forward: TGAGAATGCGAATACAAGGAGG
		Reverse: GCTAGTCGGACAAAGAACAGAA
MluLR17106	*ycf4*	Forward: ATGGAATGTAGGCAGTGGTT
		Reverse: GATACGACGAGGATAAAGACC
MluLR17402	*petE*	Forward: CATCACCTTCAAGAACAACGCC
		Reverse: ATTAGTTGACGGTGACCTTGCC
MluLR5294	*OAT4*	Forward: CAATCCTTCCAATGTCGTC
		Reverse: GGTGTAAGAACTGTCGCA
MluLR4566	*RH57*	Forward: ATAGGACGATGCGGAAGA
		Reverse: ACAGCCTGAAGATACCAACAC
MluLR17105	*rps4*	Forward: TGGCTTCAACCATTCCTG
		Reverse: TCGTTGGTTATCCTTCGTAG

### Genetic variation of candidate genes

SNP analysis showed that 19 candidate genes harboured 92 SNPs in total, with one to 17 SNPs for an individual gene (Table 5). The haplotypes of each gene were phased from SNPs, and each gene had 2–75 haplotypes and the number of genotypes based on haplotype combinations of each gene ranged from 3–71 (Table 5). The nucleotide diversity (π) ranged from 0.000304 to 0.00253 in JH, while it ranged from 0.000200 to 0.00239 in QG. The nucleotide diversity of nine and 10 genes were decreased and increased, respectively.

**Table 5.** Genetic variation of candidate genes Haplotypes are inferred from the connection of SNPs within a gene using PHASE software, and genotype of each individual is the best reconstruction of haplotypes with the highest probability. Nucleotide diversity is represented by π.

Transcripts	SNP number	Haplotype number	Genotype number	π×1000 in JH	π×1000 in QG
MluLR17106	1	2	3	0.412	0.200
MluLR2876	5	11	21	0.618	0.480
MluLR16886	4	12	15	0.509	0.500
MluLR7126	4	12	18	0.349	0.406
MluLR5294	3	5	9	0.525	0.611
MluLR12213	7	34	53	1.656	1.667
MluLR12611	7	21	26	1.693	1.727
MluLR13061	3	5	8	0.788	0.712
MluLR17624	5	15	28	1.269	1.113
MluLR16034	5	9	21	1.392	1.185
MluLR4945	5	14	26	0.747	0.932
MluLR15146	6	11	15	0.776	0.940
MluLR4148	17	75	71	2.526	2.385
MluLR18082	3	6	8	0.885	0.574
MluLR18372	5	12	23	2.157	1.488
MluLR3563	3	4	5	0.304	0.316
MluLR14116	4	8	13	0.333	0.407
MluLR18370	3	6	10	0.523	0.538
MluLR9412	2	4	4	1.650	1.697

**Fig. 6.**
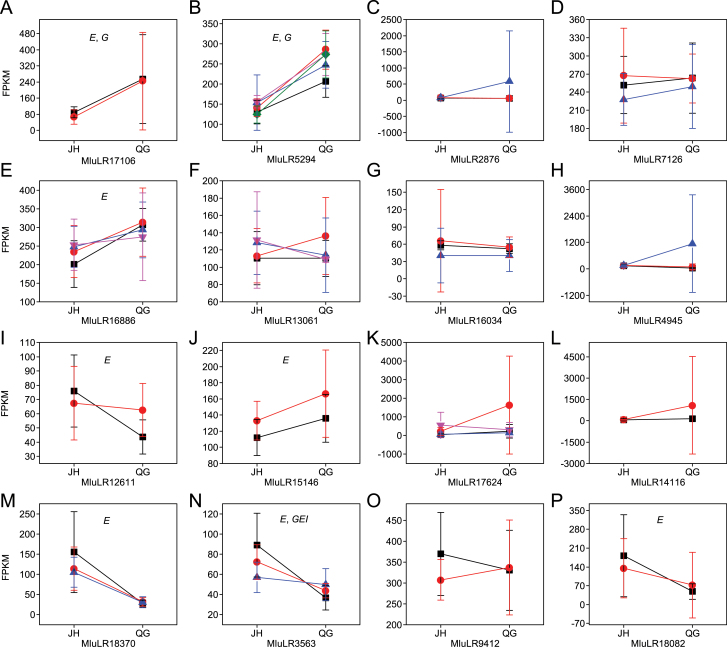
Expression reaction norms for WUE-related genes with genetic variation responding to growth environments. Detecting significant effects of those factors on the gene expression level represent, respectively, genetic variation for gene expression (*G*), phenotypic plasticity (*E*), and genetic variation for phenotypic plasticity (*GEI*, genotype-by-environment interaction). The graphs (A)–(P) represent the genes: MluLR17106 (*ycf4*), MluLR5294 (*OAT4*), MluLR2876 (*UBE3*), MluLR7126 (*SSII-2*), MluLR16886 (*WRKY4*), MluLR13061 (*LSD1*), MluLR16034 (*Cyclophilin-type PPIase*), MluLR4945 (*LCAT1-like*), MluLR12611 (*mbd106*), MluLR15146 (*FKBP-type PPIase*), MluLR17624 (*RLC*), MluLR14116, MluLR18370 (*ASRGL1-like*), MluLR3563, MluLR9412, and MluLR18082, respectively. The detailed functional annotation of these genes are given in Table 2. The average expression levels (FPKM) are computed only on genotypes with more than or equal to three individuals. Different genotypes are in a different colour and the error bars indicate the standard deviations.

The contribution of genotype, environment, and genotype×environment interaction on gene expression variation was measured using an ANOVA model for each of 19 genes with SNPs. Detecting significant effects of those factors on gene expression levels represent, respectively, genetic variation for gene expression (*G*), phenotypic plasticity (*E*), and genetic variation for phenotypic plasticity (*GEI*, genotype-by-environment interaction). Of the 19 genes, 16 with a genotype found at least three times in a field site were analysed for GEI through ANOVA (Fig. 6).

The expression levels of eight genes were not significantly affected by genotype, environment or genotype×environment interaction (Fig. 6C, D, F, G, H, K, L, O). Expression of the remaining genes were all affected by environment (*P*_E_ <0.05; Fig. 6A, B, E, I, J, M, N, P), of which five genes were affected only by environment (*P*_E_ <0.05, *P*_G_ >0.05, *P*_GEI_ >0.05; Fig. 6E, I, J, M, P). Two genes were affected by both genotype and environment (*P*_E_ <0.05, *P*_G_ <0.05, *P*_GEI_ >0.05; Fig. 6A, B). The expression of one gene was affected by genotype and environment interaction (*P*_E_ <0.05, *P*_GEI_ <0.05; Fig. 6N).

## Discussion

### The methodology of candidate gene identification

In this study, candidate genes were identified through a correlation analysis between water use efficiency and RNA-Seq data obtained from two field sites with substantially different water availability. By using the mantel test, pairwise ratios for FPKM and WUE between the two fields were calculated, which helped to exclude biases possibly caused by genetic variance and technical errors (Fig. 1). The results indicated that the matrix correlation coefficient could be effective for assessing the genetic basis of a quantitative trait based on expression data obtained from the field conditions. Thus, this method opens an opportunity for studying the genetic and genomic basis of an adaptive physiological trait using RNA-Seq data, which is becoming increasingly accessible for natural populations of organisms without reference genome sequences.

### Candidate genes involved in the improvement of WUE

Since WUE is calculated as the ratio of net photosynthesis to transpiration rate, it is expected that the candidate genes of WUE include those involved in photosynthesis. The proportion of photosynthesis genes in the whole reference transcriptome of *M*. *lutarioriparius* is about 0.8%, whereas among 48 candidates of WUE, 16.67% of them are photosynthesis-related. The photosynthetic apparatus comprises two photosystems which catalyse photosynthetic electron transport (Velthuys, 1980; Fromme and Grotjohann, 2008). PsbK, PsbI, PsbH are plastome-encoded and low molecular mass subunits that are located in the peripheral region of the photosystem II (PSII) reaction centre core (Schwenkert *et al.*, 2006; Nickelsen and Rengstl, 2013). They all play important roles in assembly and stabilizing the binding of cofactors to the PSII core (Iwai *et al.*, 2010; Pagliano *et al.*, 2013).

In sequence alignment, *psaI* and *ycf4* are both annotated in one transcript, since they are very close and are located in a large operon in the chloroplast genome. PsaI is required for the stabilization of the PsaH and PsaL subunits and they are important for building the docking platform for the light-harvesting complex of PSII (LHCII) proteins in the state transition process. Ycf4 is an assembly chaperone of photosystem I (PSI) and appears to be absolutely essential for PSI accumulation in *Chlamydomonas* (Boudreau *et al.*, 1997). In the process of photosynthesis, plastocyanin functions as an electron transfer agent between cytochrome *f* of the cytochrome *b*_6_ *f* complex and P_700_ of PSI. According to Wang *et al.* (2014a), some photosynthesis-related genes including *petE* were found to interact with a calcium-sensing receptor which accelerates stomatal movement and the formation of photosynthetic electron transport, thereby regulating WUE and drought tolerance. Thioredoxin as an integral part of the ferredoxin–thioredoxin oxidoreductase executes functions in the reductive regulation of probably hundreds of chloroplast enzymes as well as in the regeneration of components of the antioxidative system, such as peroxiredoxins (Schurmann and Buchanan, 2008). Thioredoxin-like protein is also involved in the biogenesis of the cytochrome *b*_6_ *f* complex in *Arabidposis* (Lennartz *et al.*, 2001).

All of the above genes except for MluLR15163 encoding thioredoxin-like were up-regulated in QG, which is comparable with findings that some genes in PSII were overexpressed in poplar under drought (Berta *et al.*, 2010). Chloroplast envelope membrane protein belongs to the CemA family and is possibly involved in CO_2_ uptake across the inner envelope membrane of the chloroplast (Rolland *et al.*, 1997). Pyruvate orthophosphate dikinase (PPDK) is regulated by the PPDK regulatory protein (PDRP), a bifunctional enzyme, and plays a regulatory role in the phosphoenolpyruvate (PEP)-regeneration phase of the C4 photosynthetic pathway (Naidu *et al.*, 2003; Wang *et al.*, 2008; Chastain *et al.*, 2011). Therefore, these results suggest that the up-regulation of these photosynthesis-related genes played a crucial role in improving WUE (Ruiz-Nieto *et al.*, 2015).

Because CO_2_ assimilation and transpirational water loss occur predominantly through stomatal pores, it is conceivable that genes involved in stomatal development and stomatal opening/closure affect WUE (Yoo *et al.*, 2010; Des Marais *et al.*, 2014). Among nine stomatal regulation-related genes, five encode products regulating ABA signal transduction which contain the cysteine-rich receptor-like cytosolic kinase 10 (CRK10), hydroxyacid oxidase 1, auxin response factor 4, ubiquitin-protein ligase E3, and WRKY transcription factor 4. It is well known that the plant hormone ABA is involved in abiotic stress responses and regulates stomatal closure (Lee and Luan, 2012; Lim *et al.*, 2012). CRK10 is induced by abiotic stress and up-regulated and probably negatively controls ABA signalling (Tanaka *et al.*, 2012). Hydroxyacid oxidase 1 (HAO1) could catalyse the formation of hydrogen peroxide that is involved in ABA-induced stomatal closure (Zhang *et al.*, 2001; Desikan *et al.*, 2004). Auxin response factor 4 might indirectly participate in ABA signalling due to cross-talk between auxin- and ABA-signalling under drought responses (Bianchi *et al.*, 2002). Ubiquitin-protein ligase probably positively regulates ABA signalling under abiotic stress (Zhang *et al.*, 2007) while, in *Arabidopsis*, the *CER9* gene encodes an E3 ubiquitin ligase involved in cuticle formation that could suppress transpiration and maintain plant water status (Lu *et al.*, 2012). WRKY4 was also found to be involved in ABA signalling and the mediation of stomatal closure (Rushton *et al.*, 2012).

The MluLR5858 transcript encodes the homeobox transcription factor WOX14, which might regulate stomata density and plant vascular proliferation (Nakata *et al.*, 2012; Etchells *et al.*, 2013). The MluLR5294, MluLR7126, and MluLR12213 transcripts encode anion transporter 4, starch synthase II-2, and hexose carrier protein 6 (HEX6). Anion transporter 4 is essential for resistance to abiotic stress and ion homeostasis and might be involved in the regulation of stomatal movements (Dietrich *et al.*, 2001; Wang *et al.*, 2014b). Starch synthase II-2 and HEX6 regulate the balance of starch and sucrose synthesis that can control stomata opening/closure. Thus, genes regulating stomata development and movement also played important roles in the improvement of WUE.

Twelve abiotic stress-related genes participate in multiple pathways related to abiotic stress. Drought stress responses can be regulated through epigenetic mechanisms such as DNA methylation and histone modification (Chinnusamy and Zhu, 2009; Yin *et al.*, 2014). Genes encoding the methyl-CpG binding domain 106, Mettl2-like, and LSD1 were involved in epigenetic regulation. It was found that the methyl-CpG binding domain 106 played an important role in interpreting the genetic information encoded by methylated DNA (Zou *et al.*, 2012). Mettl2 was associated with the histone deacetylase activity (Song *et al.*, 2010), and LSD1, a histone demethylase, played a role in the epigenetic regulation of gene expression (Shi *et al.*, 2004; Kim *et al.*, 2008; Papaefthimiou and Tsaftaris, 2012).

Expression of many cyclophilin and FKBP genes is induced by different abiotic stresses (Marivet *et al.*, 1992; Meza-Zepeda *et al.*, 1998; Godoy *et al.*, 2000; Sharma and Singh, 2003). P4HA1 is the subunit of prolyl 4-hydroxylase (P4H) which mediates the hydroxylation of proline, an important osmotic adjusting material greatly accumulated under drought stress and can help plants adapt to osmotic stress (Khedr *et al.*, 2003; Seki *et al.*, 2007). The up-regulation of lecithin-cholesterol acyltransferase-like 1 (LCAT1-like) under manganese toxicity may enhance the leaf concentration of sterol esters and prevent leaf senescence (Zhou *et al.*, 2013). Small heat shock protein HSP18.8, RNA helicase 57-like, ADH5-like, SRG1-like, and RLC retrotransposon were also induced by abiotic stress (de Bruxelles *et al.*, 1996; Wessler, 1996; Truesdell and Dickman, 1997; Vashisht and Tuteja, 2006).

### Expression and genetic variation of candidate genes and environmental effect

The significant correlation between qPCR validation and RNA-Seq data indicated that the relative gene expression levels between the fields are reliable, which provided a satisfactory validation of the RNA-Seq results (Li *et al.*, 2013). Although the fold changes of some candidate genes across the two sites were small, the variation in gene expression was high among individuals in each site (see Supplementary Table S2 at *JXB* online; Fig. 5). Given that all of the 48 identified genes are significantly correlated with the changed WUE, the correlation coefficient may reflect to a certain extent the relative contribution to WUE. The *psbK* gene involved in photosynthesis showed the highest contribution to WUE (*r*=0.429) (Tables 1, 2). The average correlation coefficients of photosynthesis-related, stomatal regulation-related, protein metabolism-related, abiotic stress-related, and other genes are 0.304, 0.277, 0.310, 0.280, and 0.292, respectively, which suggested that the photosynthesis- and protein metabolism- related genes showed a higher contribution to WUE than other functional categories. Although the variation range of fold change is from 0.26 to 15.60, it is noteworthy that there is no correlation between fold change of FPKM and correlation coefficients (*r*=0.043; Table 1).

The most remarkable up-regulation (15.6-fold change) is found in the MluLR10901 transcript which encodes ammonium transporter 2 (AMT2) involved in nitrogen metabolism. Leaf nitrogen is a major driver of photosynthetic capacity and is critical to determining WUE (Donovan *et al.*, 2007). The expression level of the *AMT2* gene that increased sharply in QG might imply that the significant overexpression could compensate for the high carbon assimilation rate, due to low stomatal conductance under water deficit, whereas, *psbK* and *psbI* genes were significantly up-regulated with 1.37- and 1.53-fold change, respectively (Tables 1, 3). They are elements of photosystem electron transport and might possess high WUE through steady increase of expression levels. The hierarchical clustering analysis showed strong representation of photosynthesis-related genes in cluster 3 (Fig. 4D), and half of the genes in this cluster were up-regulated more than 2-fold, which indicated a coexpression pattern of photosynthesis genes.

In Fig. 6J, MluLR15146 coding for FKBP-type PPIase was involved in abiotic stress responses since levels of FKBPs were reported to increase in response to drought stress in sorghum (Sharma and Singh, 2003). On the other hand, the expression level of MluLR12611, which encodes methyl-CpG binding protein linking DNA methylation to histone methylation (Zou *et al.*, 2012), is down-regulated under drought stress (Fig. 6I). The expression of MluLR18082 and MluLR18370 (Fig. 6M, P) classified in the ‘Other’ functional category are reduced under the water deficit environment. MluLR16886, a WRKY gene, increased its transcriptional level under abiotic stresses (Fig. 6E). WRKY transcription factors respond to several stress factors and regulate stress-related genes in order to adapt to adverse conditions (Chen *et al.*, 2012), and are also involved in ABA signalling (Rushton *et al.*, 2012). The expression level of MluLR3563 shows genetic variation for phenotypic plasticity (*P*_E_ <3E-06, *P*_GEI_=0.003), but its function is still unknown. Finally, the transcriptional levels of MluLR5294 and MluLR17106 are induced by drought stress and both show genetic variation and phenotypic plasticity (*P*_E_ <3E-16, *P*_G_=0.01; *P*_E_ <5E-05, *P*_G_=0.02). MluLR5294 codes for an anion transporter which is essential for nutrition, resistance to biotic and abiotic stresses, and stomatal movement by regulating chloride homeostasis (Dietrich *et al.*, 2001; Wang *et al.*, 2014b). MluLR17106 encodes Ycf4, an assembly chaperone of PSI and appears to be of central importance to PSI accumulation (Boudreau *et al.*, 1997; Schottler *et al.*, 2011).

The expression levels of eight genes were all affected by growth environments (Fig. 6), which indicate that they have phenotypic plasticity to adapt to the new environment. It appears unreasonable that none of the eight genes was affected by genetic variation, compared with previous studies in yeast (Smith and Kruglyak, 2008) and *Arabidopsis* (Des Marais *et al.*, 2012). One probable explanation for this phenomenon could be that the growth environment is more complicated under natural than controlled conditions. Furthermore, given that the genes are related to WUE rather than all transcripts, the WUE-related genes could have responded more sensitively to the changes of environment than to the genetic variations.

In conclusion, the five functional categories of identified genes were found relevant to the regulation of WUE, which substantiates the effectiveness of the matrix correlation analysis of physiological traits and RNA-Seq for candidate gene identification. It is noteworthy that most of the candidate genes involved in photosynthesis, stomatal regulation, and abiotic stress responses were up-regulated. Moreover, our analyses suggested that the relatively drastic changes in expression levels of the candidate genes were affected by environment rather than genotype. This study identified the candidate genes important for water deficiency adaptation of second-generation energy crops, which are subjected to further functional validation and possibly future utilization for crop improvement.

## Supplementary data

Supplementary data can be found at *JXB* online.

Supplementary Fig. S1. Distribution of CO_2_ assimilation rate (*A*) and transpiration rate (*E*) of *M*. *lutarioriparius* in Jiangxia of Hubei Province (left) and Qingyang of Gansu Province (right).

Supplementary Table S1. Collection locations, experimental field sites, and number of reads obtained for each of the sampled individuals of *Miscanthus lutarioriparius*.

Supplementary Table S2. Expression levels of 48 candidate genes among 78 individuals in Jiangxia of Hubei Province (JH) and Qingyang of Gansu Province (QG).

Supplementary Data

## References

[CIT0001] Anholt RRH Mackay TFC 2004 Quantitative genetic analyses of complex behaviours in *Drosophila* . Nature Reviews Genetics 5, 838–849. 10.1038/nrg1472 15520793

[CIT0002] Benjamini Y Hochberg Y 1995 Controlling the false discovery rate: a practical and powerful approach to multiple testing. Journal of the Royal Statistical Society, Series B (Statistical Methodology) 57, 289–300.

[CIT0003] Berta M Giovannelli A Sebastiani F Camussi A Racchi ML 2010 Transcriptome changes in the cambial region of poplar (*Populus alba* L.) in response to water deficit. Plant Biology 12, 341–354. 20398240 10.1111/j.1438-8677.2009.00320.x

[CIT0004] Bianchi MW Damerval C Vartanian N 2002 Identification of proteins regulated by cross-talk between drought and hormone pathways in *Arabidopsis* wild-type and auxin-insensitive mutants, *axr1* and *axf2* . Functional Plant Biology 29, 55–61. 10.1071/PP01113 32689451

[CIT0005] Boudreau E Takahashi Y Lemieux C Turmel M Rochaix JD 1997 The chloroplast *ycf3* and *ycf4* open reading frames of *Chlamydomonas reinhardtii* are required for the accumulation of the photosystem I complex. EMBO Journal 16, 6095–6104. 9321389 10.1093/emboj/16.20.6095 PMC1326293

[CIT0006] Boyer JS 1982 Plant productivity and environment. Science 218, 443–448. 17808529 10.1126/science.218.4571.443

[CIT0007] Carroll A Somerville C 2009 Cellulosic biofuels. Annual Review of Plant Biology 60, 165–182. 10.1146/annurev.arplant.043008.092125 19014348

[CIT0008] Cattivelli L Rizza F Badeck FW Mazzucotelli E Mastrangelo AM Francia E Mare C Tondelli A Stanca AM 2008 Drought tolerance improvement in crop plants: an integrated view from breeding to genomics. Field Crops Research 105, 1–14.

[CIT0009] Chastain CJ Failing CJ Manandhar L Zimmerman MA Lakner MM Nguyen THT 2011 Functional evolution of C_4_ pyruvate, orthophosphate dikinase. Journal of Experimental Botany 62, 3083–3091. 21414960 10.1093/jxb/err058

[CIT0010] Chen LG Song Y Li SJ Zhang LP Zou CS Yu DQ 2012 The role of WRKY transcription factors in plant abiotic stresses. Biochimica et Biophysica Acta—Gene Regulatory Mechanisms 1819, 120–128. 10.1016/j.bbagrm.2011.09.002 21964328

[CIT0011] Chen SL Renvoize SA 2006 Miscanthus. In: Wu ZY Raven PH Hong DY, eds. Flora of China . Beijing: Science Press; St Louis: Missouri Botanical Garden Press, 581–583.

[CIT0012] Chinnusamy V Zhu JK 2009 Epigenetic regulation of stress responses in plants. Current Opinion in Plant Biology 12, 133–139. 19179104 10.1016/j.pbi.2008.12.006 PMC3139470

[CIT0013] Clifton-Brown JC Lewandowski I 2000 Water use efficiency and biomass partitioning of three different *Miscanthus* genotypes with limited and unlimited water supply. Annals of Botany 86, 191–200.

[CIT0014] de Bruxelles GL Peacock WJ Dennis ES Dolferus R 1996 Abscisic acid induces the alcohol dehydrogenase gene in *Arabidopsis* . Plant Physiology 111, 381–391. 8787023 10.1104/pp.111.2.381 PMC157847

[CIT0015] de Miguel M Cabezas J-A de Maria N 2014 Genetic control of functional traits related to photosynthesis and water use efficiency in *Pinus pinaster* Ait. drought response: integration of genome annotation, allele association and QTL detection for candidate gene identification. BMC Genomics 15, 464–482. 24919981 10.1186/1471-2164-15-464 PMC4144121

[CIT0016] Des Marais DL Auchincloss LC Sukamtoh E McKay JK Logan T Richards JH Juenger TE 2014 Variation in *MPK12* affects water use efficiency in *Arabidopsis* and reveals a pleiotropic link between guard cell size and ABA response. Proceedings of the National Academy of Sciences, USA 111, 2836–2841. 10.1073/pnas.1321429111 PMC3932934 24550314

[CIT0017] Des Marais DL McKay JK Richards JH Sen S Wayne T Juenger TE 2012 Physiological genomics of response to soil drying in diverse *Arabidopsis* accessions. The Plant Cell 24, 893–914. 22408074 10.1105/tpc.112.096180 PMC3336118

[CIT0018] Desikan R Cheung MK Bright J Henson D Hancock JT Neill SJ 2004 ABA, hydrogen peroxide, and nitric oxide signalling in stomatal guard cells. Journal of Experimental Botany 55, 205–212. 14673026 10.1093/jxb/erh033

[CIT0019] Dietrich P Sanders D Hedrich R 2001 The role of ion channels in light-dependent stomatal opening. Journal of Experimental Botany 52, 1959–1967. 11559731 10.1093/jexbot/52.363.1959

[CIT0020] Donovan LA Dudley SA Rosenthal DM Ludwig F 2007 Phenotypic selection on leaf water use efficiency and related ecophysiological traits for natural populations of desert sunflowers. Oecologia 152, 13–25. 17165094 10.1007/s00442-006-0627-5

[CIT0021] Etchells JP Provost CM Mishra L Turner SR 2013 *WOX4* and *WOX14* act downstream of the PXY receptor kinase to regulate plant vascular proliferation independently of any role in vascular organisation. Development 140, 2224–2234. 23578929 10.1242/dev.091314 PMC3912870

[CIT0022] Feltus FA Vandenbrink JP 2012 Bioenergy grass feedstock: current options and prospects for trait improvement using emerging genetic, genomic, and systems biology toolkits. Biotechnology for Biofuels 5, 80. 23122416 10.1186/1754-6834-5-80 PMC3502489

[CIT0023] Fromme P Grotjohann I 2008 Structure of photosystems I and II. Results and Problems in Cell Differentiation 45, 33–72. 18066506 10.1007/400_2007_044

[CIT0024] Godoy AV Lazzaro AS Casalongue CA San Segundo B 2000 Expression of a *Solanum tuberosum* cyclophilin gene is regulated by fungal infection and abiotic stress conditions. Plant Science 152, 123–134.

[CIT0025] Hall NM Griffiths H Corlett JA Jones HG Lynn J King GJ 2005 Relationships between water-use traits and photosynthesis in *Brassica oleracea* resolved by quantitative genetic analysis. Plant Breeding 124, 557–564.

[CIT0026] Hausmann NJ Juenger TE Sen S Stowe KA Dawson TE Simms EL 2005 Quantitative trait loci affecting delta ^13^C and response to differential water availibility in *Arabidopsis thaliana* . Evolution 59, 81–96. 15792229

[CIT0027] Ings J Mur LAJ Robson PRH Bosch M 2013 Physiological and growth responses to water deficit in the bioenergy crop *Miscanthus×giganteus* . Frontiers in Plant Science 4, 1–12. 24324474 10.3389/fpls.2013.00468 PMC3839294

[CIT0028] Iwai M Suzuki T Kamiyama A Sakurai I Dohmae N Inoue Y Ikeuchi M 2010 The PsbK subunit is required for the stable assembly and stability of other small subunits in the PSII complex in the thermophilic cyanobacterium *Thermosynechococcus elongatus* BP-1. Plant and Cell Physiology 51, 554–560. 20194360 10.1093/pcp/pcq020

[CIT0029] Juenger TE McKay JK Hausmann N Keurentjes JJB Sen S Stowe KA Dawson TE Simms EL Richards JH 2005 Identification and characterization of QTL underlying whole-plant physiology in *Arabidopsis thaliana*: δ^13^C, stomatal conductance and transpiration efficiency. Plant, Cell and Environment 28, 697–708.

[CIT0030] Karaba A Dixit S Greco R Aharoni A Trijatmiko KR Marsch-Martinez N Krishnan A Nataraja KN Udayakumar M Pereira A 2007 Improvement of water use efficiency in rice by expression of *HARDY*, an *Arabidopsis* drought and salt tolerance gene. Proceedings of the National Academy of Sciences, USA 104, 15270–15275. 10.1073/pnas.0707294104 PMC1986572 17881564

[CIT0031] Khedr AHA Abbas MA Wahid AAA Quick WP Abogadallah GM 2003 Proline induces the expression of salt-stress-responsive proteins and may improve the adaptation of *Pancratium maritimum* L. to salt-stress. Journal of Experimental Botany 54, 2553–2562. 14512386 10.1093/jxb/erg277

[CIT0032] Kim JM To TK Ishida J Morosawa T Kawashima M Matsui A Toyoda T Kimura H Shinozaki K Seki M 2008 Alterations of lysine modifications on the histone H3 N-tail under drought stress conditions in *Arabidopsis thaliana* . Plant and Cell Physiolog*y* 49, 1580–1588. 18779215 10.1093/pcp/pcn133

[CIT0033] Landry CR Oh J Hartl DL Cavalieri D 2006 Genome-wide scan reveals that genetic variation for transcriptional plasticity in yeast is biased towards multi-copy and dispensable genes. Gene 366, 343–351. 16427747 10.1016/j.gene.2005.10.042

[CIT0034] Lee SC Luan S 2012 ABA signal transduction at the crossroad of biotic and abiotic stress responses. Plant, Cell and Environment 35, 53–60. 10.1111/j.1365-3040.2011.02426.x 21923759

[CIT0035] Lennartz K Plucken H Seidler A Westhoff P Bechtold N Meierhoff K 2001 *HCF164* encodes a thioredoxin-like protein involved in the biogenesis of the cytochrome *b_6_f* complex in *Arabidopsis* . The Plant Cell 13, 2539–2551. 11701887 10.1105/tpc.010245 PMC139470

[CIT0036] Li L Petsch K Shimizu R 2013 Mendelian and non-Mendelian regulation of gene expression in maize. PLOS Genetics 9. 10.1371/journal.pgen.1003202 PMC3547793 23341782

[CIT0037] Lim CW Baek W Lim S Lee SC 2012 ABA signal transduction from ABA receptors to ion channels. Genes & Genomics 34, 345–353.

[CIT0038] Liu W Mi J Song Z Yan J Li J Sang T 2014 Long-term water balance and sustainable production of *Miscanthus* energy crops in the Loess Plateau of China. Biomass and Bioenergy 62, 47–57.

[CIT0039] Liu W Sang T 2013 Potential productivity of the *Miscanthus* energy crop in the Loess Plateau of China under climate change. Environmental Research Letters 8, 1–10.

[CIT0040] Liu W Yan J Li J Sang T 2012 Yield potential of *Miscanthus* energy crops in the Loess Plateau of China. Global Change Biology Bioenergy 4, 545–554.

[CIT0041] Lu S Zhao H Des Marais DL 2012 Arabidopsis *ECERIFERUM9* involvement in cuticle formation and maintenance of plant water status. Plant Physiology 159, 930–944. 22635115 10.1104/pp.112.198697 PMC3387718

[CIT0042] Marivet J Frendo P Burkard G 1992 Effects of abiotic stresses on cyclophilin gene expression in maize and bean and sequence analysis of bean cyclophilin cDNA. Plant Science 84, 171–178.

[CIT0043] Martin B Nienhuis J King G Schaefer A 1989 Restriction fragment length polymorphisms associated with water-use efficiency in tomato. Science 243, 1725–1728. 17751282 10.1126/science.243.4899.1725

[CIT0044] Martin JA Wang Z 2011 Next-generation transcriptome assembly. Nature Reviews Genetics 12, 671–682. 10.1038/nrg3068 21897427

[CIT0045] Masle J Gilmore SR Farquhar GD 2005 The *ERECTA* gene regulates plant transpiration efficiency in *Arabidopsis* . Nature 436, 866–870. 16007076 10.1038/nature03835

[CIT0046] Meza-Zepeda LA Baudo MM Palva ET Heino P 1998 Isolation and characterization of a cDNA corresponding to a stress-activated cyclophilin gene in *Solanum commersonii* . Journal of Experimental Botany 49, 1451–1452.

[CIT0047] Mian MAR Bailey MA Ashley DA Wells R Carter TE Parrott WA Boerma HR 1996 Molecular markers associated with water use efficiency and leaf ash in soybean. Crop Science 36, 1252–1257.

[CIT0048] Mi J Liu W Yang W Yan J Li J Sang T 2014 Carbon sequestration by *Miscanthus* energy crops plantations in a broad range semi-arid marginal land in China. Science of the Total Environment 496, 373–380. 25089696 10.1016/j.scitotenv.2014.07.047

[CIT0049] Naidu SL Moose SP Al-Shoaibi AK Raines CA Long SP 2003 Cold tolerance of C_4_ photosynthesis in *Miscanthus×giganteus*: adaptation in amounts and sequence of C_4_ photosynthetic enzymes. Plant Physiology 132, 1688–1697. 12857847 10.1104/pp.103.021790 PMC167105

[CIT0050] Nakata M Matsumoto N Tsugeki R Rikirsch E Laux T Okada K 2012 Roles of the middle domain-specific *WUSCHEL-RELATED HOMEOBOX* genes in early development of leaves in *Arabidopsis* . The Plant Cell 24, 519–535. 22374393 10.1105/tpc.111.092858 PMC3315230

[CIT0051] Nei M Li WH 1979 Mathematical model for studying genetic variation in terms of restriction endonucleases. Proceedings of the National Academy of Sciences, USA 76, 5269–5273. 10.1073/pnas.76.10.5269 PMC413122 291943

[CIT0052] Nickelsen J Rengstl B 2013 Photosystem II assembly: from cyanobacteria to plants. In: Merchant SS, ed. *Annual review of plant biology* , Vol. 64 Palo Alto: Annual Reviews, 609–635. 10.1146/annurev-arplant-050312-120124 23451783

[CIT0053] Pagliano C Saracco G Barber J 2013 Structural, functional, and auxiliary proteins of photosystem II. Photosynthesis Research 116, 167–188. 23417641 10.1007/s11120-013-9803-8

[CIT0054] Papaefthimiou D Tsaftaris AS 2012 Significant induction by drought of *HvPKDM7-1*, a gene encoding a jumonji-like histone demethylase homologue in barley (*H. vulgare*). Acta Physiologiae Plantarum 34, 1187–1198.

[CIT0055] Polley HW 2002 Implications of atmospheric and climatic change for crop yield and water use efficiency. Crop Science 42, 131–140. 11756263

[CIT0056] Robson P Jensen E Hawkins S White SR Kenobi K Clifton-Brown J Donnison I Farrar K 2013 Accelerating the domestication of a bioenergy crop: identifying and modelling morphological targets for sustainable yield increase in *Miscanthus* . Journal of Experimental Botany 64, 4143–4155. 24064927 10.1093/jxb/ert225 PMC3808307

[CIT0057] Rolland N Dorne AJ Amoroso G Sultemeyer DF Joyard J Rochaix JD 1997 Disruption of the plastid *ycf10* open reading frame affects uptake of inorganic carbon in the chloroplast of *Chlamydomonas* . EMBO Journal 16, 6713–6726. 9362486 10.1093/emboj/16.22.6713 PMC1170276

[CIT0058] Ruiz-Nieto JE Aguirre-Mancilla CL Acosta-Gallegos JA Raya-Perez JC Piedra-Ibarra E Vazquez-Medrano J Montero-Tavera V 2015 Photosynthesis and chloroplast genes are involved in water-use efficiency in common bean. Plant Physiology and Biochemistry 86, 166–173. 25500453 10.1016/j.plaphy.2014.11.020

[CIT0059] Rushton DL Tripathi P Rabara RC 2012 WRKY transcription factors: key components in abscisic acid signalling. Plant Biotechnology Journal 10, 2–11. 21696534 10.1111/j.1467-7652.2011.00634.x

[CIT0060] Saeed AI Sharov V White J et al J 2003 TM4: A free, open-source system for microarray data management and analysis. Biotechnique*s* 34, 374–378. 12613259 10.2144/03342mt01

[CIT0061] Sang T 2011 Toward the domestication of lignocellulosic energy crops: learning from food crop domestication. Journal of Integrative Plant Biology 53, 96–104. 21261812 10.1111/j.1744-7909.2010.01006.x

[CIT0062] Sang T Zhu W 2011 China’s bioenergy potential. Global Change Biology Bioenergy 3, 79–90.

[CIT0063] Schmittgen TD Zakrajsek BA Mills AG Gorn V Singer MJ Reed MW 2000 Quantitative reverse transcription-polymerase chain reaction to study mRNA decay: comparison of endpoint and real-time methods. Analytical Biochemistry 285, 194–204. 11017702 10.1006/abio.2000.4753

[CIT0064] Schottler MA Albus CA Bock R 2011 Photosystem I: its biogenesis and function in higher plants. Journal of Plant Physiology 168, 1452–1461. 21255865 10.1016/j.jplph.2010.12.009

[CIT0065] Schurmann P Buchanan BB 2008 The ferredoxin/thioredoxin system of oxygenic photosynthesis. Antioxidants & Redox Signaling 10, 1235–1273. 18377232 10.1089/ars.2007.1931

[CIT0066] Schwenkert S Umate P Dal Bosco C Volz S Mlcochova L Zoryan M Eichacker LA Ohad I Herrmann RG Meurer J 2006 PsbI affects the stability, function, and phosphorylation patterns of photosystem II assemblies in tobacco. The Journal of Biological Chemistry 281, 34227–34238. 16920705 10.1074/jbc.M604888200

[CIT0067] Seki M Umezawa T Urano K Shinozaki K 2007 Regulatory metabolic networks in drought stress responses. Current Opinion in Plant Biology 10, 296–302. 17468040 10.1016/j.pbi.2007.04.014

[CIT0068] Sharma AD Singh P 2003 Comparative studies on drought-induced changes in peptidyl prolyl cis-trans isomerase activity in drought-tolerant and susceptible cultivars of *Sorghum bicolor* . Current Science 84, 911–918.

[CIT0069] Shi YJ Lan F Matson C Mulligan P Whetstine JR Cole PA Casero RA Shi Y 2004 Histone demethylation mediated by the nuclear amine oxidase homolog LSD1. Cell 119, 941–953. 15620353 10.1016/j.cell.2004.12.012

[CIT0070] Smith EN Kruglyak L 2008 Gene–environment interaction in yeast gene expression. PLOS Biology 6, 810–824. 10.1371/journal.pbio.0060083 PMC2292755 18416601

[CIT0071] Sohlenkamp C Shelden M Howitt S Udvardi M 2000 Characterization of *Arabidopsis* AtAMT2, a novel ammonium transporter in plants. FEBS Letters 467, 273–278. 10675553 10.1016/s0014-5793(00)01153-4

[CIT0072] Somerville C Youngs H Taylor C Davis SC Long SP 2010 Feedstocks for lignocellulosic biofuels. Science 329, 790–792. 20705851 10.1126/science.1189268

[CIT0073] Song YA Wu KQ Dhaubhadel S An LZ Tian LN 2010 *Arabidopsis* DNA methyltransferase AtDNMT2 associates with histone deacetylase AtHD2s activity. Biochemical and Biophysical Research Communications 396, 187–192. 20331964 10.1016/j.bbrc.2010.03.119

[CIT0074] Stephens M Donnelly P 2003 A comparison of Bayesian methods for haplotype reconstruction from population genotype data. American Journal of Human Genetics 73, 1162–1169. 14574645 10.1086/379378 PMC1180495

[CIT0075] Suyker AE Verma SB 2012 Gross primary production and ecosystem respiration of irrigated and rainfed maize–soybean cropping systems over 8 years. Agricultural and Forest Meteorology 165, 12–24.

[CIT0076] Tanaka H Osakabe Y Katsura S Mizuno S Maruyama K Kusakabe K Mizoi J Shinozaki K Yamaguchi-Shinozaki K 2012 Abiotic stress-inducible receptor-like kinases negatively control ABA signaling in *Arabidopsis* . The Plant Journal 70, 599–613. 22225700 10.1111/j.1365-313X.2012.04901.x

[CIT0077] Tardieu F 2012 Any trait or trait-related allele can confer drought tolerance: just design the right drought scenario. Journal of Experimental Botany 63, 25–31. 21963615 10.1093/jxb/err269

[CIT0078] Teulat B Merah O Sirault X Borries C Waugh R This D 2002 QTLs for grain carbon isotope discrimination in field-grown barley. Theoretical and Applied Genetics 106, 118–126. 12582879 10.1007/s00122-002-1028-8

[CIT0079] Thumma BR Naidu BP Chandra A Cameron DF Bahnisch LM Liu CJ 2001 Identification of causal relationships among traits related to drought resistance in *Stylosanthes scabra* using QTL analysis. Journal of Experimental Botany 52, 203–214. 11283164

[CIT0080] Trapnell C Williams BA Pertea G Mortazavi A Kwan G van Baren MJ Salzberg SL Wold BJ Pachter L 2010 Transcript assembly and quantification by RNA-Seq reveals unannotated transcripts and isoform switching during cell differentiation. Nature Biotechnology 28, 511–515. 10.1038/nbt.1621 PMC3146043 20436464

[CIT0081] Truesdell GM Dickman MB 1997 Isolation of pathogen/stress-inducible cDNAs from alfalfa by mRNA differential display. Plant Molecular Biology 33, 737–743. 9132065 10.1023/a:1005728420374

[CIT0082] Vashisht AA Tuteja N 2006 Stress responsive DEAD-box helicases: a new pathway to engineer plant stress tolerance. Journal of Photochemistry and Photobiology B-Biology 84, 150–160. 10.1016/j.jphotobiol.2006.02.010 16624568

[CIT0083] Velthuys BR 1980 Mechanisms of electron flow in Photosystem II and toward Photosystem I. Annual Review of Plant Physiology 31, 545–567.

[CIT0084] Wallace JS 2000 Increasing agricultural water use efficiency to meet future food production. Agriculture Ecosystems & Environment 82, 105–119.

[CIT0085] Wang DF Portis AR Moose SP Long SP 2008 Cool C_4_ photosynthesis: pyruvate Pi dikinase expression and activity corresponds to the exceptional cold tolerance of carbon assimilation in *Miscanthus×giganteus* . Plant Physiology 148, 557–567. 18539777 10.1104/pp.108.120709 PMC2528129

[CIT0086] Wang WH Chen J Liu TW Chen J Han AD Simon M Dong XJ He JX Zheng HL 2014 *a* . Regulation of the calcium-sensing receptor in both stomatal movement and photosynthetic electron transport is crucial for water use efficiency and drought tolerance in *Arabidopsis* . Journal of Experimental Botany 65, 223–234. 24187420 10.1093/jxb/ert362 PMC3883291

[CIT0087] Wang YZ Hills A Blatt MR 2014 *b* . Systems analysis of guard cell membrane transport for enhanced stomatal dynamics and water use efficiency. Plant Physiology 164, 1593–1599. 24596330 10.1104/pp.113.233403 PMC3982726

[CIT0088] Wessler SR 1996 Plant retrotransposons: turned on by stress. Current Biology 6, 959–961. 8805314 10.1016/s0960-9822(02)00638-3

[CIT0089] Xu Q Xing S Zhu C 2015 Population transcriptomics reveals a potentially positive role of expression diversity in adaptation. Journal of Integrative Plant Biology 57, 284–299. 25251542 10.1111/jipb.12287

[CIT0090] Xue GP McIntyre CL Chapman S Bower NI Way H Reverter A Clarke B Shorter R 2006 Differential gene expression of wheat progeny with contrasting levels of transpiration efficiency. Plant Molecular Biology 61, 863–881. 16927201 10.1007/s11103-006-0055-2

[CIT0091] Yan J Chen WL Luo F 2012 Variability and adaptability of *Miscanthus* species evaluated for energy crop domestication. Global Change Biology Bioenergy 4, 49–60.

[CIT0092] Yan J Zhu C Liu W Luo F Mi J Ren Y Li J Sang T 2015 High photosynthetic rate and water use efficiency of *Miscanthus lutarioriparius* characterize an energy crop in the semiarid temperate region. Global Change Biology Bioenergy 7, 207–218.

[CIT0093] Yin HF Chen CJ Yang J 2014 Functional genomics of drought tolerance in bioenergy crops. Critical Reviews in Plant Sciences 33, 205–224.

[CIT0094] Yoo CY Pence HE Jin JB Miura K Gosney MJ Hasegawa PM Mickelbart MV 2010 The *Arabidopsis* GTL1 transcription factor regulates water use efficiency and drought tolerance by modulating stomatal density via transrepression of *SDD1* . The Plant Cell 22, 4128–4141. 21169508 10.1105/tpc.110.078691 PMC3027182

[CIT0095] Zhang X Zhang L Dong FC Gao JF Galbraith DW Song CP 2001 Hydrogen peroxide is involved in abscisic acid-induced stomatal closure in *Vicia faba* . Plant Physiology 126, 1438–1448. 11500543 10.1104/pp.126.4.1438 PMC117144

[CIT0096] Zhang YY Yang CW Li Y Zheng NY Chen H Zhao QZ Gao T Guo HS Xie Q 2007 SDIR1 is a RING finger E3 ligase that positively regulates stress-responsive abscisic acid signaling in *Arabidopsis* . The Plant Cell 19, 1912–1929. 17573536 10.1105/tpc.106.048488 PMC1955734

[CIT0097] Zhang Z Xu P Shao H Liu M Fu Z Chu L 2011 Advances and prospects: biotechnologically improving crop water use efficiency. Critical Reviews in Biotechnology 31, 281–293. 21486183 10.3109/07388551.2010.531004

[CIT0098] Zhou CP Qi YP You X Yang LT Guo P Ye X Zhou XX Ke FJ Chen LS 2013 Leaf cDNA-AFLP analysis of two citrus species differing in manganese tolerance in response to long-term manganese-toxicity. BMC Genomics 14, 1–19. 24034812 10.1186/1471-2164-14-621 PMC3847489

[CIT0099] Zou XQ Ma W Solov’yov IA Chipot C Schulten K 2012 Recognition of methylated DNA through methyl-CpG binding domain proteins. Nucleic Acids Research 40, 2747–2758. 22110028 10.1093/nar/gkr1057 PMC3315304

