# Supplementary material for: Transcriptome-wide characterization of candidate genes for improving the water use efficiency of energy crops grown on semiarid land
Source: J Exp Bot. 2015 Jul 13;66(20):6415–29. doi: 10.1093/jxb/erv353 (PMC4588889; doi:10.1093/jxb/erv353)
Supplement: Supplementary Data [file supp_66_20_6415__index.html]

Transcriptome-wide characterization of candidate genes for improving the water use efficiency of energy crops grown on semiarid land — Transcriptome-wide characterization of candidate genes for improving the water use efficiency of energy crops grown on semiarid land — Supplementary Data 

# Transcriptome-wide characterization of candidate genes for improving the water use efficiency of energy crops grown on semiarid land

## Supplementary Data

Data files

- Supplementary Data - Supplementary Data
- Supplementary Data - Supplementary Data
