# Supplementary material for: Transcriptome-wide characterization of candidate genes for improving the water use efficiency of energy crops grown on semiarid land
Source: J Exp Bot. 2015 Jul 13;66(20):6415–29. doi: 10.1093/jxb/erv353 (PMC4588889; doi:10.1093/jxb/erv353)
Supplement: Supplementary Data [file supp_erv353_jexbot154070_file002.pdf]

Transcriptome-wide characterization of candidate genes for improving water use efficiency of energy crops grown on semiarid land

Yangyang Fan, Qian Wang, Lifang Kang, Wei Liu, Qin Xu, Shilai Xing, Zhihong Song, Caiyun Zhu, Cong Lin, Juan Yan, Jianqiang Li, Tao Sang

Journal of Experimental Botany

Supplementary Data

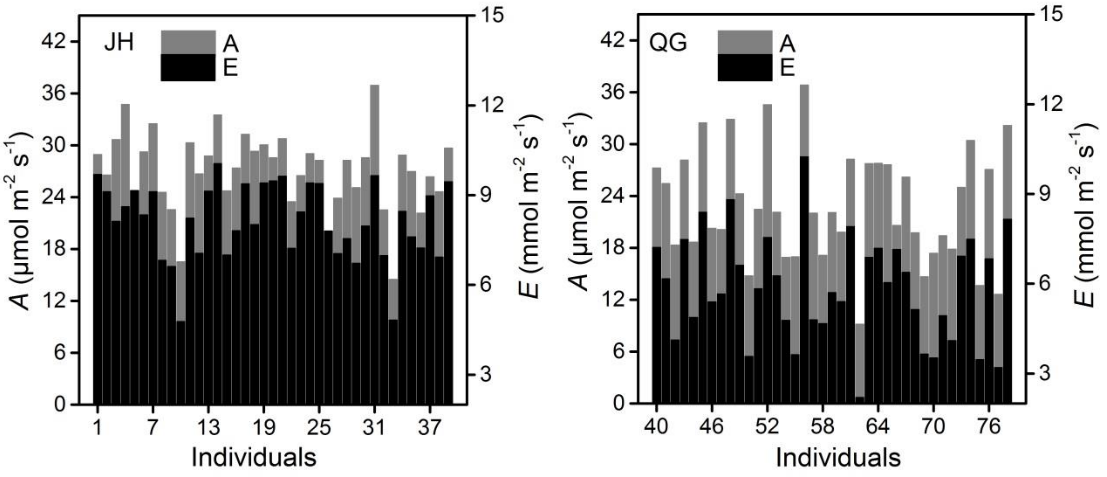

Figure S1 Distribution of CO_2 assimilation rate (*A*) and transpiration rate (*E*) of *M. lutarioriparius* in Jiangxia of Hubei Province (left) and Qingyang of Gansu Province (right). The individuals are ordered by the populations collecting from native habitats, which can be acquired from Supplementary Table S1. The *A* and *E* of each individual are presented as gray bar and black bar, respectively.

Supplementary Table S1. Collection locations, experimental field sites, and number of reads obtained for each of the sampled individuals of *Miscanthus lutarioriparius*.

**Collection location**	**JH**				**QG**			
	**Individual**	**Raw reads**	**Filtered reads**	**No.**	**Individual**	**Raw reads**	**Filtered reads**	**No.**
	WQ2	43686498	33618379	1	WQ5	23590976	20525591	40
Wuqiqu, Hubei Prov.	WQ1	30173536	25235534	2	WQ6	27086792	23088318	41
	WQ3	31537820	25286855	3				
	PZ1	45150386	38364788	4	PZ1	45150386	38364788	42
Pazidang, Hubei Prov.	PZ2	32792150	28544472	5	PZ5	30269368	26649124	43
	PZ3	28001176	21188859	6	PZ6	37960688	31147497	44
	LJ3	45345498	37552766	7	LJ6	32456888	26102513	45
Lijiamen, Hubei Prov.	LJ2	43919030	36117592	8	LJ2	43919030	36117592	46
	LJ1	43323444	35334561	9	LJ1	43323444	35334561	47
	YP2	27512128	20772266	10	YP4	22641646	20130944	48
Yanpucun, Hubei Prov.	YP1	31198938	24679664	11	YP6	23165520	19184013	49
					YP5	86048676	64802980	50
	ZJ2	43552040	28390547	12	ZJ5	32662294	26490551	51
Zhoujiatai, Hubei Prov.	ZJ3	36071716	28171130	13	ZJ4	34958110	28054790	52
					ZJ3	36071716	28171130	53
	FX3	33005164	26062880	14	FX2	47390224	38102534	54
Fuxingcun, Hubei Prov.	FX2	47390224	38102534	15	FX6	30222028	24676698	55
					FX5	33572492	27385997	56
	BF3	34052136	23540539	16	BF6	30362906	22939969	57
Baofeng, Hubei Prov.	BF1	45572396	36600553	17	BF4	35274742	30594116	58
	BF2	56914796	46091572	18	BF2	56914796	46091572	59

	BH2	35725706	30269125	19	BH6	31315492	25559154	60
Beihecun, Hubei Prov.	BH3	34996070	24856871	20	BH5	27553940	22347259	61
	BH1	52524532	44565337	21	BH4	36889662	29273083	62
	XS2	39261792	32155826	22	XS5	38630470	31422960	63
Xiaoshajiao, Hubei Prov.	XS1	45335982	38726003	23	XS4	32214572	27744349	64
	XS3	29461846	21521519	24	XS3	29461846	21521519	65
	DQ2	29675498	25626432	25	DQ4	24586640	21039933	66
Daqiao, Hunan Prov.	DQ1	35114046	29995273	26	DQ3	33896624	24732915	67
	DQ3	33896624	24732915	27	DQ5	31983770	27685163	68
	BS1	38421936	28016967	28	BS6	54695418	41859279	69
Baishaxiang, Hunan Prov.	BS3	33099308	22164647	29	BS4	26528830	21864284	70
	BS2	32190424	26934678	30	BS5	21323672	16324437	71
	JS1	36912454	30984766	31	JS4	38014442	31745772	72
Junshanqu, Hunan Prov.	JS2	45441470	38752164	32	JS5	44670564	36538013	73
	JS3	60536184	50685108	33				
	ZZ3	52032478	43887401	34	ZZ3	52032478	43887401	74
Zhanjiaju, Hunan Prov.	ZZ2	47515874	37968661	35	ZZ5	32948474	28517012	75
	ZZ1	32948384	25853844	36				
	LZ2	43790696	36522200	37	LZ5	33085734	26991865	76
Liangzihu, Hubei Prov.	LZ3	25962024	20724327	38	LZ3	25962024	20724327	77
	LZ1	70068378	59940432	39	LZ4	26005084	22505395	78

Individual names consist of two capital letters representing a population name abbreviated from the collection locations followed by individual number in the population, except for the ZZ (Zhanjiaju, Hunan Prov.).

Supplementary Table S2. Expression levels of 48 candidate genes among 78 individuals in Jiangxia of Hubei Province (JH) and Qingyang of Gansu Province (QG).

FPKM (JH) and FPKM (QG) values represent the average expression levels of each transcript in the experimental fields in Jiangxia of Hubei Province (JH) and Qingyang of Gansu Province (QG), respectively. FPKM (QG/JH) values represent the fold change of expression levels of each transcript, which were calculated as the ratio of FPKM (QG) to FPKM (JH).
